# Fractal kinetic characteristics of hard-rock uranium leaching with sulfuric acid

**DOI:** 10.1098/rsos.180403

**Published:** 2018-09-19

**Authors:** Sheng Zeng, Jinzhu Li, Kaixuan Tan, Shuwen Zhang

**Affiliations:** Nuclear Resources Engineering College, University of South China, Hengyang, Hunan 421001, People's Republic of China

**Keywords:** hydrometallurgy, uranium ores, leaching kinetics, fractal

## Abstract

In order to study the fractal dynamic properties of uranium leach mining and discuss the influence of ore crushing on the dynamics of leach mining, uranium mine ore rocks in southern China were selected as the research object and an acid leaching experiment was performed on the ore samples with different fractal dimensions of 1.1, 1.4, 1.7, 2.0, 2.3 and 2.6. In the column leaching experiment, a PVC pipe with an inner diameter of 112 mm and a height of 1500 mm was used. The uranium content was determined by using titanium trioxide that was placed into a 0.1 mg ml^−1^ standard uranium solution, and a sampling rate of once daily with a 5 ml volume of leaching solution was adopted after 8 h drenching time. The results show that the flow rate of the leaching solution depends on the distribution of the ore's particle size, that is, a larger fractal dimension results in a smaller flow rate. The concentration of the uranium leaching solution reaches a maximum value which subsequently decreases with time on the third day of the experiment, and it seems that the changes in the uranium concentration tend to be stable at around 15 days. Moreover, the concentration seems to increase with the increasing fractal dimension, and the fractal dimension of the ore particle size has a significant impact on the leaching kinetics. When the fractal dimension is between 1.1 and 2.6, the uranium dissolution rate, *K*, increases with the increasing fractal dimension. The kinetic reaction of the uranium leaching is a liquid–solid one, which is controlled by chemical reactions in the earlier phase. While the middle reaction phase is mainly chemical-diffusion reaction coupling, and the latter part of the reaction is controlled by diffusion. As the fractal dimension increases, the liquid–solid reaction controlled by diffusion appears at earlier phases. When the fractal dimension is greater than 2.0, the time of entering the diffusion control phase only changed little with the increasing of the fractal dimension. At last, a fractal dimension of 2.0 is suggested for the acid leaching of uranium ore crushing.

## 1. Introduction

At present, leach mining is a new type of deposit mining technology that includes a solid–liquid transfer process transmitting useful elements from the ore to the leaching solution. Leach mining is cost-effective, the uranium ore can be put into production quickly, it is a simple operation technology and easily managed, the ecological environment impacts and exploitation of low-grade mineral resources is minimal. Therefore, the study and application of this technology are of interest to domestic and foreign scholars. Especially, the leach mining of uranium has occurred in many countries, such as the USA, Australia, Russia, China and Iran. Compared with the traditional methods of uranium mining, the method now used for uranium mining has greatly improved, and the handbooks and research reports of *in situ* leaching of uranium have been published by the International Atomic Energy Agency [1,2].

The dissolution of metal in leach mining is influenced by various factors, such as texture, voids, particle fractal dimension and permeability. In order to improve the leaching rate effectively, the permeability of the mining layer is a key factor, which is related to the successful development of leach mining. As uranium resources are widely used, the demand for uranium has greatly increased, and the effective mining of uranium ore is the primary task for uranium miners. In the case of uranium leach mining, the required temperatures are low and the chemical and diffusion reactions are slow; hence, it is difficult to achieve equilibrium. Leach mining production often depends on the reaction rate, which is related not only to the concentration of the leaching liquid but also to the movement of seepage in the ore-bearing rock. Understanding seepage related to the leaching solution requires an understanding of fluid mechanics, where kinetic conditions greatly influence the leach mining process. The main purpose of studying the kinetics of leaching was to reveal the control mechanism during leaching. Thus, targeted measures are taken to strengthen the leaching rate. Consequently, the study of leaching kinetics has become one of the most important topics in the research of leach mining technology, where many scholars have achieved encouraging results [3–19]. However, there are relatively few studies on the dynamic characteristics of uranium leaching. Based on the distribution of the fractal dimension of Mellado *et al*. [20], Song Jian-bin *et al*. [20] improved their dynamic model of ore heap leaching and established a uranium ore heap leaching fractal dynamic model that was verified by column leaching experiments, and their results were better than those of the aforementioned study. The influence of fractal distribution on uranium leaching was studied by Zeng Sheng *et al*. [22,23], who showed that the shape and size of different rock particles had their own specific fractal characteristics, and the fractal dimension had a strong relationship with the leaching rate. A sandstone uranium deposit in the Yili basin was studied by Tan Kai-xuan *et al*. [24,25], and the results showed that the geological geochemical characteristics of ore had a significant influence on uranium leaching dynamics, and the effects of the uranium leaching reaction rate were mainly controlled by diffusion, while the influence of surface chemical reactions was relatively small. Liu Yu-long *et al*. [26] used a JMA (Johnson–Mehl–Avrami) dynamics model to analyse the dynamic characteristics of the acidification and bacterial-leaching processes, and they discovered that the initial reaction process was controlled by diffusion, the intermediate reaction process was mixed controlled and the later reaction rate was close to zero. Under the conditions of different fractal dimensions, Zhao He-Yong *et al*. [27] performed a dynamic experiment, and they found that the dissolution rate increased with the increasing fractal dimension, they also fitted the corresponding fractal dynamic equation to experimental results for further analysis.

In this work, we studied the acid leaching of hard rock in different fractal dimensions based on fractal theory, and the leaching kinetic equations for different fractal dimensions and a general leaching kinetics equation were established. The results suggested that a fractal dimension of 2.0 was the optimum value for the dissolution of uranium deposits.

## 2. Ore samples and experiment

### 2.1. Sampling and sample processing

The ore samples were from a uranium deposit in Jiang-xi, China, this mine is a granite-type uranium deposit, which is generally called as hard-rock uranium deposit, and it belongs to a medium–low T hydrothermal ore deposit. Its mineralization age is 47–94 Ma, and an internal or external contact zone of rock bodies produces the uranium deposit. Red fragmentation alters the rock deposits in the southeastern Huanggao district, and green fragmentation changes rock deposits in the Niu-wei ridge and Feng-shu district in the north. The ore-hosting rocks contain the composite batholith, and the lithology belongs to a shallow, metamorphic sandy slate of the Cambrian Longshan group. The rock is hard and tight, and its colour is grey to grey-black. The ore samples were taken from throughout the mine laneway, and an ore body boundary grade of 0.035% was determined with a *γ* radiometer. Working face was determined using a 0.5 × 0.5 m network whose ore body grade is 0.121%. The chemical components of the uranium ores were tested by the chemical reaction method in our study. According to the geological data, the samples selected for the experiment are granite-type uranium, and the content of carbonate is low. Because the reactions in the process of uranium leaching are mainly the redox ones, several chemical components that may have an influence on the experiment were tested selectively. From the results of chemical analysis of the uranium ores, the derived chemical components are shown in table 1.

**Table 1.** Chemical components of uranium ores.

chemical components	CaO	MgO	FeO	Al_2_O_3_	MnO	SiO_2_	Fe_2_O_3_	U	U(IV)	U(VI)	others
percentage content (%)	0.24	0.37	0.77	10.78	0.07	75.79	1.00	0.134	0.0646	0.0694	10.85

For the uranium column leaching experiment, a PVC pipe with an inner diameter of 112 mm and a height of 1500 mm was used. Our study showed that the ratio of the inner pipe diameter to particle size was an important factor impacting the leaching process, and such effects could be avoided when the ratio was between 4 and 20. Based on the established experimental scheme, and the experimental condition and facilities, the ratio was set to around 5; i.e. the diameter of the PVC pipe is 112 mm and the maximum particle size of ore sample is less than 28 mm. Therefore, the ore samples were crushed and then sieved (using ASTM standard sieves) to given particles with size fractions of 0.15–0.4, 0.4–0.63, 0.63–0.9, 0.9–4.0, 4.0–6.9, 6.9–9.0 mm.

Fractal theory is one of the nonlinear mathematic methods and has been used extensively to describe irregular and fragmented objects with no characteristic length. The crushed ores with irregular geometry have self-similar properties, and the particle size distribution always manifested as a fractal distribution throughout the leaching process. Hence, the number of rock fragments could be determined statistically, and a distribution regularity of particle size was studied using the sieving method. According to the research of literature [28–29], a fractal model for the particle size distribution originally put forward by Mandelbrot was adopted in this study [30], as shown in the following equation:

2.1 $$\frac{M(r)}{M(r_{0})}={\left(\frac{r}{r_{\max}}\right)}^{3-D}.$$

where *M*(*r*) is the quality of the rock block whose diameter is less than *r*, *M*(*r*_0_) is the total quality of all rock blocks, *r*_max_ is the maximum diameter of the fractured ore and *D* is the fractal dimension of the particle size distribution.

According to the requirements of the designed test device, the fractured ore quality of every fractal dimension is 5000 g. Based on equation (2.1), the qualities needed by the different fractal dimension of the different particle size ores are obtained. Admittedly, the amount of leached uranium increases with the degree of rock fragmentation. However, a column leaching experiment was adopted in our study, we consider not only the impact of degree of rock fragmentation but also the influence of permeability of crushed ores on the leaching efficiency and the amount of leached uranium in the process of the leaching experiment. In general, the porosity and the permeability of crushed ores reduce with the degree of rock fragmentation increasing, which are adverse to the uranium leaching. In our study, six groups of samples were sieved to given fractal dimensions of 1.1, 1.4, 1.7, 2.0, 2.3 and 2.6 and labelled with the numbers, no.1, no.2, … , no.6, respectively (table 2).

**Table 2.** Particle size distribution of ores corresponding to different fractal dimension (unit: g).

		particle size (mm)						
experimental samples	fractal dimension	0–0.15	0.15–0.40	0.40–0.63	0.63–0.90	0.90–4.00	4.00–6.90	6.90–9.00
no.1	1.1	2.1	11.4	18.5	31.0	1008.1	1946.9	1982.0
no.2	1.4	7.1	27.2	36.7	54.6	1240.5	1902.3	1731.6
no.3	1.7	24.4	62.9	70.3	93.0	1491.7	1797.3	1460.4
no.4	2.0	83.3	138.9	127.8	150.0	1722.2	1611.1	1166.7
no.5	2.3	284.6	280.9	211.7	220.4	1836.7	1317.1	848.6
no.6	2.6	972.1	467.0	286.8	264.7	1624.4	880.9	504.1

### 2.2. Measurement of the leaching solution flow rate

In the process of the leach mining, the main ore flow pattern arising from the heaping of the leaching solution is natural percolation in a gravity field. In order to study the effects of the fractal dimension distribution on the leaching solution flow rate, the following test was performed. Crushed ores with different fractal particle size dimensions were put into a PVC pipe with a diameter of 100 mm and height of 500 mm, and the loading height was 250 mm. Then, clear water was gradually poured into the pipe until the water level reached the upper port.

In order to keep the hydrostatic pressure invariant in the leaching process, we continuously maintained the water level so that it was always filled up to the top of the barrel mouth. At last, the solution flow rate of different crushed ores with given fractal dimensions is shown in table 3. It can be concluded that a larger fragment distribution fractal dimension results in a lower flow rate. The reason for this is that the seepage velocity seriously depends on the porosity of the ore fragments, that is, the larger the fractal dimension, the properties of crushed ores with lower porosity and permeability may be generated; and the lower the porosity, the slower the flow rate.

**Table 3.** Different fractal dimension corresponding to flow rate.

dimension	1.1	1.4	1.7	2.0	2.3	2.6
flow rate (mm s^−1)^	2.3	2.2	2.1	2.0	0.9	0.8

### 2.3. The choice of solution leaching reagent and the measurement of uranium

Based on the chemical composition of uranium ores (table 1), the main composition is formed from silicates, and the ore rock solution leaching reagent meets the need of the acid leaching method, i.e. the low price of the H_2_SO_4_ and the convenient transportation, hence H_2_SO_4_ solution was used as the solution leaching reagent. The H_2_SO_4_ solution with the concentration of 1.84 g l^−1^ and the purity of 98% was made into a leaching solution with the concentration of 20 g l^−1^.

Hydrochloric acid, U_3_O_8_ and hydrogen peroxide were mixed into the standard uranium solution with the concentration of 0.1 mg ml^−1^, and the titration method of titanium trioxide was used to estimate the uranium content. Firstly, a 1 ml standard uranium solution was extracted and placed in a 150 ml conical flask, then 12 ml of phosphoric acid and 8 ml of water were added. Subsequently, the titanium trichloride was dropped into the solution until the tested solution turned into a stable purple red colour by continuously shaking in a ventilated kitchen. Then two drops of sodium nitrite were added, and the solution was continuously shaken until the colour disappeared; next, we dropped 5 ml of urea into the solution immediately and kept shaking the solution until bubbles disappeared, and then three drops of sodium diphenylamine sulfonate were added. Finally, the solution was titrated with the appropriate concentration of ammonium vanadate standard solution until it presented a slightly purplish red for 30 s. Based on the following equation (2.2), the titre of the ammonium vanadate standard solution to uranium was calculated, and the percentage content of uranium was calculated based on equation (2.3):

2.2 $$T=\frac{C_{1}\times V_{1}}{V_{2}}.$$

where *T* is the titre of the ammonium vanadate standard solution to uranium (mg ml^−1^), *C*_1_ is the concentration of the uranium standard solution (mg ml^−1^), *V*_1_ is the volume of the uranium standard solution (ml) and *V*_2_ is the volume of consumed ammonium vanadate standard solution (ml).

2.3 $$C_{2}=\frac{T\times V_{3}}{V_{4}},$$

where *T* is the titre of the ammonium vanadate standard solution to uranium (mg ml^−1^), *V*_3_ is the volume of consumed ammonium vanadate standard solution (ml), *V*_4_ is the volume of the sample weighting (ml) and *C*_2_ is the concentration of the uranium (mg ml^−1^).

## 3. Results and discussion

### 3.1. Dissolution processes

According to the analysis results of the main chemical components of the experimental samples, the valence states of uranium are mainly tetravalent uranium and hexavalent uranium. However, it is difficult to leach tetravalent uranium with sulfuric acid. Generally, the tetravalent uranium is first oxidized to hexavalent uranium for leaching and extraction. During the leaching process, uranium is transferred into the leaching solution in the form of uranyl ion $\left(\text{U}{\text{O}}_{2}^{2+}\right)$ forming uranyl sulfate (UO_2_SO_4_) and uranyl sulfate anion complex ($\text{U}{\text{O}}_{2}{\left(\text{S}{\text{O}}_{4}\right)}_{2}^{2-}$, $\text{U}{\text{O}}_{2}{\left(\text{S}{\text{O}}_{4}\right)}_{2}^{4-}$). As shown in table 1, the ore sample contains considerable ferrous iron and ferric iron. The ferric iron has strong oxidizing properties and can oxidize tetravalent uranium into hexavalent uranium. When the sulfuric acid leaching is used, oxygen in the air can be acted as an oxidant and can not only oxidize tetravalent uranium into hexavalent uranium but also oxidize ferrous iron into ferric iron. The detailed chemical reactions are as follows:

3.1 $$\text{4FeO}+{\text{O}}_{2}+6{\text{H}}_{2}\text{S}{\text{O}}_{4}=2\text{F}{\text{e}}_{2}{\left(\text{S}{\text{O}}_{4}\right)}_{3}+6{\text{H}}_{2}\text{O, }$$

3.2 $$\text{F}{\text{e}}_{2}{\text{O}}_{3}+3{\text{H}}_{2}\text{S}{\text{O}}_{4}=\text{F}{\text{e}}_{2}{\left(\text{S}{\text{O}}_{4}\right)}_{3}+3{\text{H}}_{2}\text{O, }$$

3.3 $$\text{U}{\text{O}}_{2}+2\text{F}{\text{e}}^{3+}={\text{UO}}_{2}^{2+}+2\text{F}{\text{e}}^{2+},$$

3.4 $$\text{2U}{\text{O}}_{2}+{\text{O}}_{2}=2\text{U}{\text{O}}_{3},$$

3.5 $$\text{U}{\text{O}}_{3}+2{\text{H}}^{+}=\text{U}{\text{O}}_{2}^{2+}+{\text{H}}_{2}\text{O, }$$

3.6 $$\text{U}{\text{O}}_{2}^{2+}+\text{S}{\text{O}}_{4}^{2-}=\text{U}{\text{O}}_{2}\text{S}{\text{O}}_{4},$$

3.7 $$\text{U}{\text{O}}_{2}\text{S}{\text{O}}_{4}+\text{S}{\text{O}}_{4}^{2-}=\text{U}{\text{O}}_{2}{\left(\text{S}{\text{O}}_{4}\right)}_{2}^{2-}$$

3.8 $$\;\text{and}\;\text{U}{\text{O}}_{2}{\left(\text{S}{\text{O}}_{4}\right)}_{2}^{2-}+\text{S}{\text{O}}_{4}^{2-}=\text{U}{\text{O}}_{2}{\left(\text{S}{\text{O}}_{4}\right)}_{3}^{4-}.$$

Sulfuric acid leaching is a non-selective process. During the leaching process, mineral impurities are also dissolved in the leaching solution, and those minerals exist in the solution in ionic state or precipitate produced by plasma in combination with sulfuric acid root. The main chemical reaction is as follows:

3.9 $$n\mathrm{Si}O_{2}+nH_{2}O\overset{H_{2}SO_{4}}{⟶}[H_{2}Si_{3}{]}_{n}.$$

Under acidic conditions, the polysilicic acid formed by the reaction is transferred into the leachate in a colloidal state, which may block the pore passage of the ore and adhere to the surface of the ore particles, causing difficulty in later leaching.

3.10 $$\text{A}{\text{l}}_{2}{\text{O}}_{3}+3{\text{H}}_{2}\text{S}{\text{O}}_{4}=A{\text{l}}_{2}{\left(\text{S}{\text{O}}_{4}\right)}_{3}+3{\text{H}}_{2}\text{O}.$$

This reaction is unlikely to occur in a sulfuric acid solution, the solubility usually does not exceed 3–5% of its contents in the ores.

3.11 $$\text{CaO + }{\text{H}}_{2}\text{S}{\text{O}}_{4}=\mathrm{CaS}{\text{O}}_{4}+{\text{H}}_{2}\text{O}$$

and

3.12 $$\text{MgO + }{\text{H}}_{2}\text{S}{\text{O}}_{4}=\mathrm{MgS}{\text{O}}_{4}+{\text{H}}_{2}\text{O}.$$

The calcium–magnesium compound can react with dilute sulfuric acid easily by forming calcium sulfate and magnesium sulfate. However, calcium sulfate and magnesium sulfate have low solubility and are easy to reprecipitate or adhere to the surface of the particles, and then easy to block tiny channels in the sample, which have a greater impact on subsequent uranium leaching.

As mentioned above, during the process of acid leaching of uranium, not only the leaching process of uranium but also the reaction of other chemical components contained in uranium ore in the leaching process should be considered. The chemical components not only consume acid but also form precipitate and reprecipitate that have a great influence on the middle and late stages of leaching. In particular, the precipitated and reprecipitated materials may block the micropores and cause immersion dead angle (leaching blind area). Moreover, the reprecipitated materials may adhere to the surface of the ore particles forming as the wrapped state, and then the leaching solution can no longer migrate and diffuse into the interior of the ores, thereby the leaching rate decreases.

### 3.2. Testing and discussion of the uranium concentration in the leaching solution

**Figure 1.**
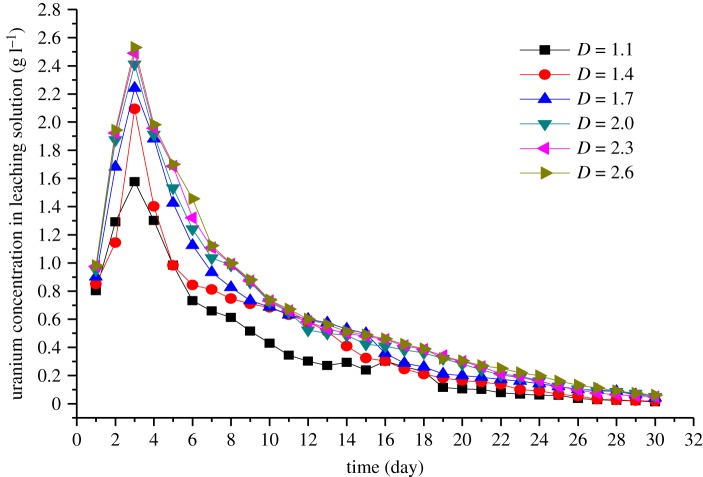
The relationship between uranium leaching concentration and time.

**Figure 2.**
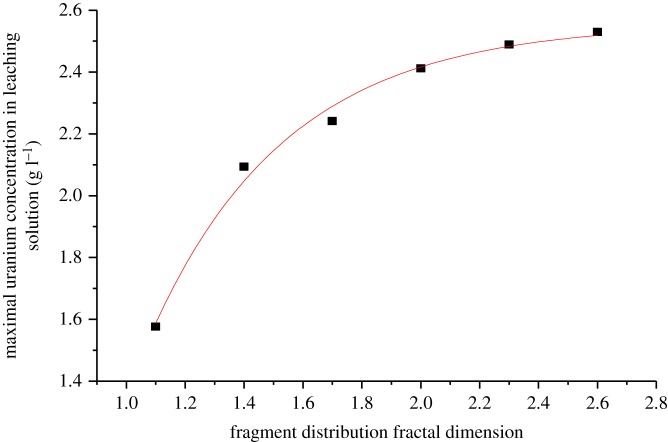
The relationship between maximal uranium concentration and fractal dimension.

The column leaching experiment was performed in a PVC pipe, and oxygen in the air was used as an oxidant in the experiment. A spraying time with 8 h every day was adopted to pour the leaching solution into the uranium ores, while the reaction between the leaching solution and ores was kept in the remaining 16 h and oxygen in the air can enter into the cracks of the ore acting as the oxidant. Sampling of the leaching solution was used with 5 ml per day, and the solution should be diluted due to the high uranium concentration. After it was diluted five times, 5 ml diluted solution was used to test the concentration of the uranium. Finally, the relationship between the uranium concentration and the leaching time (figure 1), and the relationship between the fractal dimension and the maximum uranium concentration in the leaching solution were obtained (figure 2).

It can be seen from figure 1 that the uranium concentration in the leaching solution reaches a maximum value on the third day after leaching, and then a continuous decrease in the uranium concentration in the leach solution occurs. The reason is that the uranium on the surface of the ore rock is nearly dissolved within 3 days, and afterwards, the leaching solution continues to disperse and is transported into the interior of the ore rock. In addition, the leaching process in the first 3 days changes the pore structure of the ore rock, probably due to the precipitation of the leaching process that makes the pores smaller, and the flow rate of the infiltration solution may lead to the migration of smaller particles into the narrow pores where the seepage pathway in the ores is blocked, which leads to decrease in the uranium concentration of the leaching solution rapidly after 3 days. However, the decreasing rate becomes flat after 15 days because the uranium metal near to the surface is dissolved thoroughly after 15 days, and the dispersion and transportation of the leaching solution became stable at that time resulting in the relatively constant uranium concentration of the leaching solution.

It can be seen from figure 2 that the maximum uranium concentration increases with the increasing fractal dimension of the particle size of ores. One reason for this phenomena is the increased fractal dimension result in the increasing weight of smaller particle size ores in the tested samples. Therefore, the specific surface area of the ore increases, and then the contact surface of the ore with the leaching solution also increases, so that the dissolution of uranium becomes more plentiful. Based on the chemical composition for different uranium ore particle sizes, the other reason may be the high grade of uranium in the smaller particle size of the ores. The content of uranium in the tested samples increases with the increasing fractal dimension. In addition, it can also be seen from figure 2 that when the fractal dimension is less than 2.0, an obvious rise in the concentration of leaching solution can be seen. However, when the fractal dimension is larger than 2.0, the change is moderate. The reason may be that the ratio of the small size ores to the large size ores increase with the increasing fractal dimension. As analysed above, it can be concluded that the fractal dimension of 2.0 can be considered as an initial index to determine the size changes of the particle ores.

### 3.3. Reaction kinetics

The process of metal leaching belongs to a liquid–solid reaction in nature. With respect to the study of the liquid–solid reaction kinetics, many different mathematical models of kinetic reactions, such as the unreacted core shrinking model, the particle model, have been proposed. One of the most important models is the unreacted core model, which has been successfully and extensively used. Based on the research results of Z. Ekinci [7], the uranium ore leaching fractal dynamics can be studied with the unreacted core model [31] and the reaction is shown as follows:

3.13 $${\text{A}}_{\left(\mathrm{fluid}\right)}+{\text{B}}_{\left(\mathrm{solid}\right)}\to \text{Products}.$$

If the action is controlled by a chemical reaction, the reaction kinetics is given as follows:

3.14 $$1-{\left(1-X\right)}^{1/3}=K_{1}t.$$

where *X* is the ratio of the accumulated amount of the leached uranium to the total content of uranium in the ores, and *K* is the uranium dissolution rate (g d^−1^).

If the action is controlled by diffusion through a metal-ore surface, the reaction kinetics equation can be written as follows:

3.15 $$1-3{\left(1-X\right)}^{2/3}+2(1-X)=K_{2}t.$$

**Figure 3.**
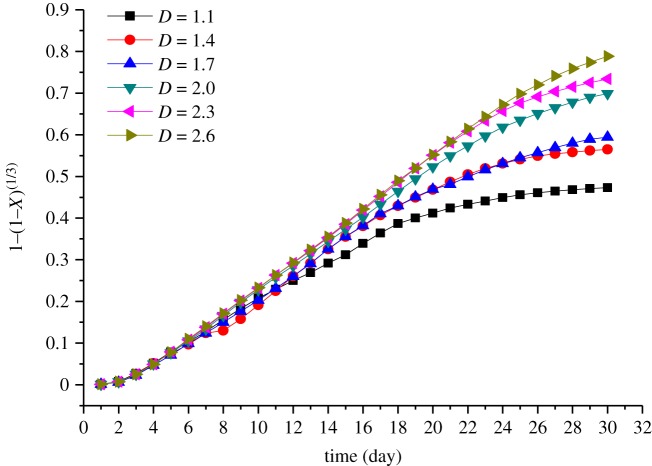
Plots of 1 − (1 − *X*)^1/3^ versus time.

**Figure 4.**
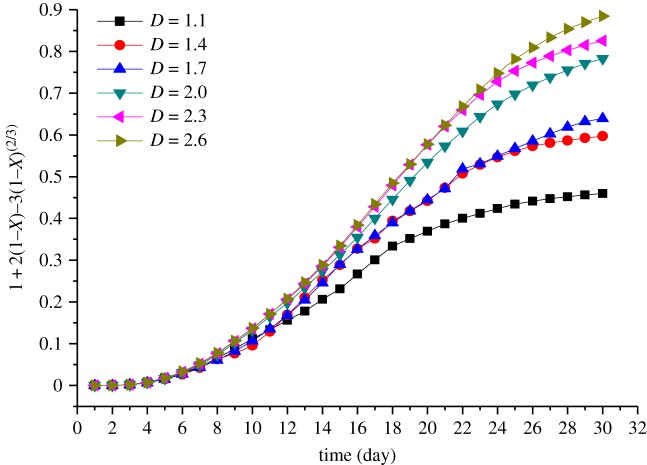
Plots of 1 − 3(1 − *X*)^2/3^ + 2(1 − *X*) versus time.

Applying a regression analysis to the tested data by using these equations, it is found that the rate of the uranium metal dissolution is controlled by the chemical reaction and the diffusion reaction. Based on the leaching results of the six ore samples, the integrated rate values of the leaching for every tested sample are described by equations (3.14) and (3.15) and shown in figures 3 and 4, respectively.

### 3.4. Discussion and analysis

Figure 3 shows the integrated rate values of the leaching kinetic for the ores with different fractal dimension based on the chemical reaction control model. The reaction rate reaches a maximum value after 3 days, where the curve increased linearly initially, and the length of the straight line increases with the increasing fractal dimension. However, the slopes of the curves are almost unchanged after 15 days for the tested samples whose fractal dimensions are lower than 2.0. Subsequently, the slopes begin to decrease, but when the fractal dimension is larger than 2.0, the time of this decreasing is delayed by 4–5 days. The reason for this is that the ratio of the small size ores increases by the increasing fractal dimension, as well as the specific surface area, and then the reaction time of uranium leaching increases. Finally, the effect of chemical reaction on the ore surface and the dissolution rate of uranium leaching become weak gradually as the content of uranium in the ores decreases.

Figure 4 shows the integrated rate values of the leaching kinetic for the ores with different fractal dimensions based on the diffusion reaction control model. During the first 4 days of the experiment, the integrated rate is almost zero. After 5–7 days, the trend of the curve was basically the same as that under the control of the chemical reaction in figure 3. The reason for this phenomenon may be that the initial reaction, surface chemical reaction, mainly occurs on the surface of the ores and the leaching solution has not yet entered into the core of the ores. It indicates that the diffusion reaction does not work and may have a certain extent of the inhibitory effect. As the reaction goes on, the diffusion reaction rate gradually promotes. After 5–20 days, although the role of the diffusion reaction control gradually increases, this phase is still dominated by the chemical reaction control. Hence, as the reaction time increases, the chemical reaction on the ore surface is completed gradually and the molecules of the leaching solution diffuse inward following the ‘unreacted core principle.’ In addition, the chemical reactions are accompanied by heat releasing. According to the Brownian motion laws, the degree of molecular motion and collision are more aggravated. Then, after approximately 20 days, the effect of the surface diffusion control is stronger than that of the chemical reaction control, and the surface diffusion control starts to dominate the effect on uranium leaching. Besides, it is also possible that as the leaching time goes on, the content of uranium decreases and the pore structure of the ore changes, and the rate of the chemical reaction is relatively slow. Meanwhile, the movement of molecular collision is acute, causing the reaction transfer from the surface to the ‘core' of the ore.

Figures 3 and 4 show that the slope of the curves begins to slow down after 15 days when the fractal dimension of ores is lower than 2.0, while when the fractal dimension is greater than or equal to 2.0, the time of this decreasing is delayed by 4–5 days. The reason for this phenomenon may be that the amount of small particles in the samples gradually decreases with decreasing fractal dimension. The smaller the fractal dimension, the faster the flow rate of the immersion solution. As the reaction is proceeding, the surface chemical reaction in ores whose fractal dimension is less than 2.0 is basically completed after 15 days, and the previous leaching causes not only the change in the particle size but also the precipitation. When the fractal dimension is less than 2.0, the large particle ores account for a large proportion and the smaller particle ores can move down through the gap in the sample, resulting in formation of the majority of large particles in the upper part of the sample. Then, the porosity and the permeability of the sample both become higher as well as the flow rate of the leaching solution in the upper position, causing a reduction in the reaction time of the leaching. In addition, the percolation pathway and the dominant flow may be formed in the tested samples, as the high porosity of the sample and high flow rate of the leaching solution. Thus, the dead zone may be generated in the sample that the leaching solution cannot reach. Meanwhile, some smaller particle ores driven by the liquid may further migrate to the lower position, then the area of the dead angle may increase and the permeability may decrease in the lower position as certain particle ores may block the passage of the liquid. Thus, the slope of the curve, with a fractal dimension less than 2.0, changes slowly with the passage of time. The sample with fractal dimension greater than 2.0 has relatively uniform particle ores and less dead zone, consequently, the leaching solution has stable flow rate and the slope of the leaching kinetic curve becomes steady after 15 days.

**Figure 5.**
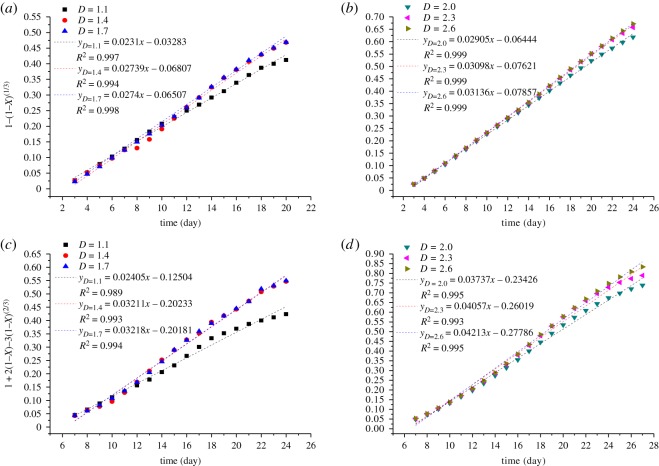
The kinetic curve of uranium leaching based on different models. (*a*,*b*) Chemical reaction control; (*c*,*d*) diffusion reaction control.

The linear segment slope of the curve in figures 3 and 4 reflects the dissolution rate, *K*, of the uranium in the experiment. The values of *K* shown in figure 5 are obtained by linear fitting in the linear segment of the curve, whereby *K* is substituted into equations (3.14) and (3.15), and the leaching dynamic equation of different fractal dimensions of the tested samples is also obtained.

**Figure 6.**
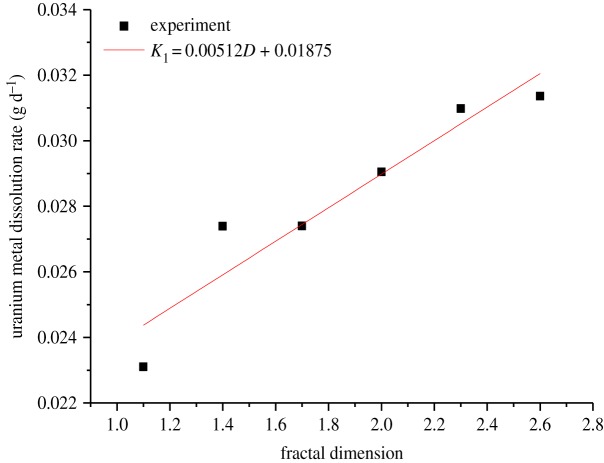
Plot of *K*_1_ versus fractal dimension.

It can be seen from figure 6 that *K*_1_ and *D* approximately meet the linear relations, and the linear relations of *K*_1_ and D are given as follows:

3.16 $$K_{1}=0.00512D+0.01875\;(1.0<D<2.6).$$

When the equation is substituted in equation (3.14), the fractal dynamic equation of uranium is given as follows:

3.17 $$1-3{\left(1-X\right)}^{1/3}=(0.00512D+0.01875)t\;(1.0<D<2.6).$$

**Figure 7.**
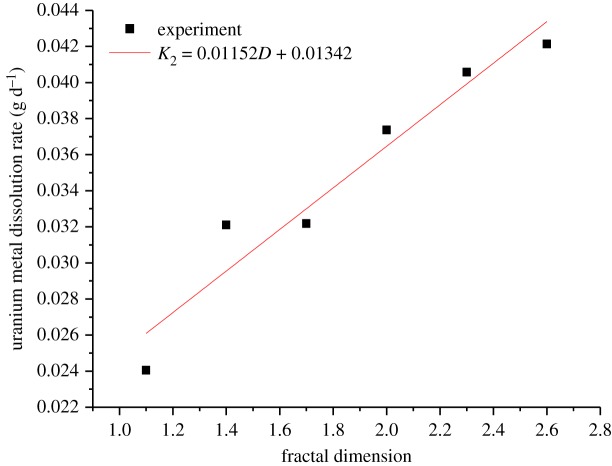
Plot of *K*_2_ versus fractal dimension.

It can be seen from figure 7 that *K*_2_ and *D* approximately also meet the linear relations, and the linear relation between *K*_2_ and *D* is given as follows:

3.18 $$K_{2}=0.01152D+0.01342\;(1.0<D<2.6).$$

When equation (3.18) is substituted in equation (3.15), the fractal dynamic equation of uranium can be obtained as follows:

3.19 $$1-3{\left(1-X\right)}^{2/3}+2(1-X)=(0.01152D+0.01342)t\;(1.0<D<2.6).$$

Figures 6 and 7 reflect the relationship between the dissolution rate of uranium and the different fractal dimensions of ore samples, that is, the dissolution rate increases with the increasing fractal dimension. According to the fitting results (equations (3.16) and (3.18)), the slope of the dissolution rate curve under the chemical reaction control is smaller than that of the dissolution rate curve under the surface diffusion reaction control at the same fractal dimension. The reason may be that with the increasing fractal dimension, heavy load of the small particle size ores in the tested samples results in the increase in specific surface area, which brings about much more change, for example, the ores contact with the leaching solution. Thus, the speed of the uranium dissolution reaction increases, and the chemical reaction control occurs much earlier than the diffusion reaction control in the leaching experiment. However, as the chemical reaction goes on, the heat of the chemical reaction has been accumulated and the movement of the molecular collision has been aggravated, then the leaching solution diffuses into the nucleus of the ores gradually; afterwards, the diffusion reaction rate also increases gradually.

**Figure 8.**
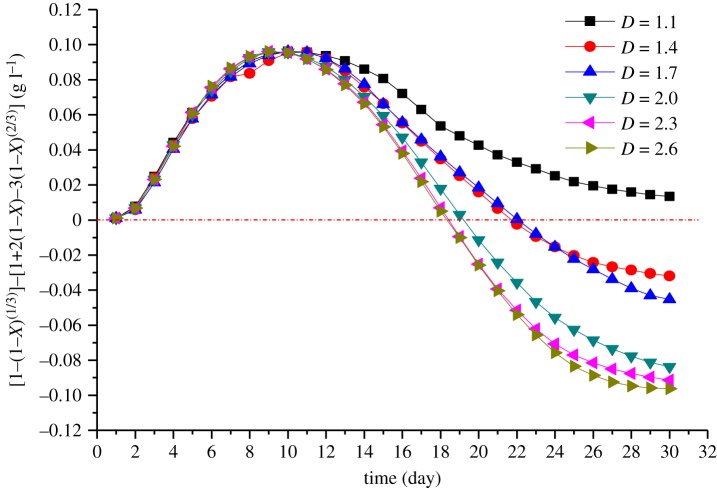
Plots of (*K*_1_*t* – *K*_2_*t*) versus time.

As can be seen from figure 8, the dominance of the chemical reactions increases during the first 11 days. After 11 days, the dominant position of the chemical reaction begins to decrease. This phase corresponds to the coupling of the chemical-diffusion reaction. In the next 20 days, the chemical reaction stays at the same position as the diffusion reaction. From our experimental data, it is seen that the dominance of the chemical reactions decreases between 11 and 20 days, whereby the effect of the diffusion reaction gradually becomes obvious. Although the chemical reaction is still dominant at this time, the diffusion reaction could not be ignored at this stage. With the increasing fractal dimension, the dominance of diffusion begins sooner. The reason for this phenomenon is that with the increasing fractal dimension, the content of small size ores increases as well as the surface area, and as a faster surface chemical reaction rate occurred, the reaction time is greatly reduced and the surface chemical reaction is completed sooner. Thus, the dominance of the diffusion control phase becomes much earlier when the fractal dimension increases. At about 11 days, the leaching begins to enter the coupling stage of the chemical-diffusion reaction. As shown in figures 3 and 4, both the chemical reaction rate and the diffusion reaction rate reach the maximum stable state. At this time, the higher the dissolution rate, the earlier the chemical-diffusion reaction coupling phase. Ultimately, the leaching time of the mid-term chemical-diffusion reaction coupling phase can be obviously shortened and the diffusion reaction control phase can arrive earlier, and finally, the leaching rate is higher. Because the chemical reaction on the surface of the ore is basically completed in the pre-metaphase stage, it can be considered that the ore with a fractal dimension of less than 2.0 should be re-crushed to increase the specific surface area of the ore, reduce the particle size and shorten the migration path of the target element, so as to increase the leaching rate, shorten the leaching time and improve the leaching efficiency. Figure 8 also shows that when the fractal dimension is larger than or equal to 2.0, as the fractal dimension increases, the time when the diffusion control phase dominates changes very little. The reason may be that the content of small size ores increases as the fractal dimension increases. Although the chemical reaction rate is promoted, the decreasing porosity of ores limits the flow rate of the leaching solution. The chemical reaction rate is related to the concentration of the leaching solution, so when the concentration of the leaching solution is constant, the reaction rate increases with increasing reactants. However, when the amount of reactants exceeds a certain value, the reaction rate will keep unchanged, and there is even a negative feedback effect. In summary, a fractal dimension of 2.0 is suggested for the acid leaching of uranium ore crushing.

## 4. Conclusion

From the results of our study, the following conclusions were obtained:

(1) The uranium ore samples are leached by an H_2_SO_4_ solution with the concentration of 20 g l^−1^. When the fractal dimension is between 1.1 and 2.6, the uranium dissolution rate, *K*, increases with the increasing fractal dimension, but the time for entering the diffusion control phase decreases. When the fractal dimension is larger than or equal to 2.0, the uranium dissolution rate and the time taken to enter the diffusion control phase change little. Therefore, it can be concluded that the optimum fractal dimension for uranium ore dissolution is 2.0, which may be considered as a reference for the particle size choice for the heap leaching and *in situ* blasting and leaching. In addition, based on the results obtained from a kinetic formula of the chemical reaction control and the diffusion reaction control, the assumption is made that if the phase of the chemical-diffusion coupling arrives sooner, the leaching time would be greatly shortened and the leaching rate would increase. This conclusion may provide a theoretical basis for the study of the leaching mining through kinetics.
(2) The reaction rate of uranium metal reaches the maximum value on the third reaction day, where the curve increases linearly and the curvature of the straight line becomes smaller with the decreasing uranium content in the leaching experiment. The participation of surface diffusion reaction is low and may even have a negative feedback effect on the leaching before 4 days. The reaction rate of the uranium metal increases gradually after 5 days and reaches the maximum value after some days. As the content of uranium metal in the ore sample decreases, the reaction rate begins to decrease significantly. However, this rate is still larger than that of the chemical reaction.
(3) Our study shows that the reaction kinetics of uranium leaching is a liquid–solid reaction, where the pre-reaction is controlled by the chemical reaction. Then, the middle of the reaction is mainly chemical-diffusion reaction coupling, and the latter part of the reaction is controlled by diffusion. Besides, the fractal dynamic equation of uranium leaching in this paper can provide a theoretical basis for the development of solution mining technology.

## Supplementary Material

Experimental raw data

## Data accessibility

Experimental data have been uploaded as part of the electronic supplementary material.

## References

[1] IAEA. 2001 *Manual of acid in situ leach uranium mining technology*. IAEA-TECDOC-1239. Vienna, Austria: IAEA.

[2] IAEA. 2004 *Recent developments in uranium resources and production with emphasis on in situ leach mining*. IAEA-TECDOC-1396. Vienna, Austria: IAEA.

[3] Huo J, Song H, Du J, Guan Q 2015 Coupled fluid flow and chemical dissolution model based on surface reaction and mass transfer control in a rough fracture. Chin. J. Rock Mech. Eng. 34, 1013–1021.

[4] Wu Z, Zhu Y 2015 Kinetics of enargite chemical leaching. Chin. J. Rare Met. 39, 735–740.

[5] Kang XY, Su Y, Zhao YL, Zha ZT 2012 Study on Fe leaching kinetic in the acid solution process of yellow phosphorus slag. Bull. Chin. Ceram. Soc. 31, 1367–1370.

[6] Liyu Z, Shaoming Y, Bin L, Liang C, Lingli H 2011 Kinetics of leaching vanadium with alkaline from stone coal. Chin. J. Rare Met. 35, 101–105.

[7] Ekinci Z, Colak S, Cakici A, Sarac H 1998 Leaching kinetics of sphalerite with pyrite in chlorine saturated water. Miner. Eng. 11, 279–283. (10.1016/S0892-6875(98)00006-5)

[8] Liao Y, Zhou J, Huang F, Wang Y 2015 Leaching kinetics of calcification roasting calcinate from multimetallic sulfide copper concentrate containing high content of lead and iron. Sep. Purif. Technol. 149, 190–196. (10.1016/j.seppur.2015.05.042)

[9] Zhong SP 2015 Leaching kinetics of gold bearing pyrite in H_2_SO_4_–Fe_2_(SO_4_)_3_ system. Trans. Nonferrous Met. Soc. Chin. 25, 3461–3466. (10.1016/S1003-6326(15)63983-8)

[10] Baghalha M 2012 The leaching kinetics of an oxide gold ore with iodide/iodine solutions. Hydrometallurgy 113–114, 42–50. (10.1016/j.hydromet.2011.11.013)

[11] Martínez-Luévanos A, Rodríguez-Delgado MG, Uribe-Salas A, Carrillo-Pedroza FR, Osuna-Alarcón JG 2011 Leaching kinetics of iron from low grade kaolin by oxalic acid solutions. Appl. Clay Sci. 51, 473–477. (10.1016/j.clay.2011.01.011)

[12] Behera SS, Parhi PK 2016 Leaching kinetics study of neodymium from the scrap magnet using acetic acid. Sep. Purif. Technol. 160, 59–66. (10.1016/j.seppur.2016.01.014)

[13] Wang HH, Li GQ, Zhao D, Ma JH, Yang J 2017 Dephosphorization of high phosphorus oolitic hematite by acid leaching and the leaching kinetics. Hydrometallurgy 171, 61–68. (10.1016/j.hydromet.2017.04.015)

[14] Yang L-J, Chen NC, Zhong XP, Xie QL, Gao J, Lan YX, Liu CM, Wu ZY 2015 Kinetics analysis of leaching lead from lead residue in NaCl-HCl solution. Chin. J. Nonferrous Met. 25, 1705–1712.

[15] Liao Y, Peng Z, Zhou J, Huang F 2015 Research on kinetics of leaching of arsenic from dust containing high arsenic. J. Sichuan Univ. (Eng. Sci. Ed.) 47, 200–206.

[16] Yang B, Yang T, Xu Z, Liu H, Shi W, Yang X 2018 Numerical simulation of the free surface and water inflow of a slope, considering the nonlinear flow properties of gravel layers: a case study. R. Soc. open sci. 5, 172109 (10.1098/rsos.172109) 29515904 PMC5830793

[17] Qi MF, Zheng YF, Gui SL 2010 Kinetic study on leaching lead from waste lead-acid batteries for lead plaster chloride system. Min. Metall. Eng. 30, 61–64.

[18] Liu ZH, Cao ZY, Liu ZY, Li QH, Li YH 2012 Leaching kinetics of willemite in (NH_4_)_2_SO_4_-NH_3_-H_2_O system at higher mass ratio of liquid to solid. J. Cent. South Univ. (Sci. Technol.) 43, 418–423.

[19] Weiyi S, Ding Sanglan SS 2011 Leaching kinetics of Mn from low grade pyrolusite with SO_2_ in liquid phase. J. Sichuan Univ. (Eng. Sci. Ed.) 43, 199–203.

[20] Mellado ME, Cisternas LA, Gálvez ED 2009 An analytical model approach to heap leaching. Hydrometallurgy 95, 33–38.

[21] Song JB, Ding DX, Ye YJ, Li GY, Fu HY, Hu N, Wang YD 2014 Fractal kinetic model for heap leaching of uranium ore. At. Energy Sci. Technol. 48, 598–603.

[22] Zeng S, Tan KX 2011 Effect of fractal feature of the crushing size distribution on uranium leaching rate. Min. Metall. Eng. 31, 16–18.

[23] Zeng S, Tan KX, Sang X, Shi WG 2011 Numerical simulation on multi-field and multi-process coupling dynamics of in-situ leaching of uranium. At. Energy Sci. Technol. 45, 500–505.

[24] Liang C, Tan KX, Jiang L, Yanshi X, Wei H, Qiang M 2013 Characteristics of leaching kinetics of a sandstone uranium deposit in Yili basin. Metal Mine 43, 18–20+38.

[25] Jiang WS, Tan KX, Xie YS, Hu Y 2014 The influence of geologic feature of ores on kinetics of uranium leaching from some granite uranium deposit in South China. J. Univ. South China (Sci. Technol.) 28, 39–45.

[26] Liu YL, Li GY, Wang YT, Hu N, Yu Q, Ma L, Ding DX 2018 Kinetic analysis of uranium ore in acidification and bacteria column leaching. At. Energy Sci. Technol. 52, 227–234.

[27] Zhao H, Li L, Zeng G, Liu Y 2013 Fractal kinetics of uranium leaching. Min. Technol. 13, 31–32+67.

[28] Hu XF, Hu DW 2007 Correlation of particle size distribution fractal dimension with powder fluidity and flow addition reagent action effect. J. Mater. Sci. Eng. 25, 205–510.

[29] Ni HY, Liu XL 2008 Grain-size distribution and fractal structures of solid grains in debris-flow deposits. Sediment. Geol. Tethyan Geol. 28, 35–41.

[30] Chan A, Tuszynski JA 2016 Automatic prediction of tumour malignancy in breast cancer with fractal dimension. R. Soc. open sci. 3, 160558 (10.1098/rsos.160558) 28083100 PMC5210682

[31] Que W, Tan Y, Zeng Y, Wang S 2002 Geochemical kinetics and mass transport of in-situ uranium leaching. Beijing, China: Atomic Energy Press.

