# Supplementary material for: Fractal kinetic characteristics of hard-rock uranium leaching with sulfuric acid
Source: R Soc Open Sci. 2018 Sep 19;5(9):180403. doi: 10.1098/rsos.180403 (PMC6170527; doi:10.1098/rsos.180403)
Supplement: Experimental raw data [file rsos180403supp1.doc]

**One. Experimental sample preparation data**

The experimental samples are sieved to obtain particles of different particle sizes. Then according to the requirement of different fractal dimension ratios, samples of the six component dimensions D = 1.1, 1.4, 1.7, 2.0, 2.3, 2.6 were prepared in the following table. The total mass of each set of samples was 5000.0 g.

The quality distribution of different fractal dimensions (unit: g)

| Sample serial number | Fractal dimension D | Particle size /mm | | | | | | | Total mass/g |
| --- | --- | --- | --- | --- | --- | --- | --- | --- | --- |
|  |  | 0-0.15 | 0.15-0.40 | 0.40-0.63 | 0.63-0.90 | 0.90-4.00 | 4.00-6.90 | 6.90-9.00 |  |
| 1 | 1.1 | 2.1 | 11.4 | 18.5 | 31.0 | 1008.1 | 1946.9 | 1982.0 | 5000.0 |
| 2 | 1.4 | 7.1 | 27.2 | 36.7 | 54.6 | 1240.5 | 1902.3 | 1731.6 | 5000.0 |
| 3 | 1.7 | 24.4 | 62.9 | 70.3 | 93.0 | 1491.7 | 1797.3 | 1460.4 | 5000.0 |
| 4 | 2.0 | 83.3 | 138.9 | 127.8 | 150.0 | 1722.2 | 1611.1 | 1166.7 | 5000.0 |
| 5 | 2.3 | 284.6 | 280.9 | 211.7 | 220.4 | 1836.7 | 1317.1 | 848.6 | 5000.0 |
| 6 | 2.6 | 972.1 | 467.0 | 286.8 | 264.7 | 1624.4 | 880.9 | 504.1 | 5000.0 |

**Two.** **Leaching experiment data**

Column leaching experiments were performed using the prepared experimental samples. There were a total of 6 groups. The six groups of samples had fractal dimension values of D=1.1, 1.4, 1.7, 2.0, 2.3, and 2.6. The total mass of the experimental sample in each group was 5000.0 g. The uranium concentration was measured by titanium trioxide titration in the experiment, and the test cycle was measured once a day for 30 days.

Sample 1 （D=1.1）

| Time/Day | Total volume of leachate/ml | Uranium concentration/g/l |
| --- | --- | --- |
| 1 | 500 | 0.802 |
| 2 | 495 | 1.291 |
| 3 | 490 | 1.576 |
| 4 | 495 | 1.301 |
| 5 | 498 | 0.984 |
| 6 | 490 | 0.731 |
| 7 | 495 | 0.659 |
| 8 | 500 | 0.613 |
| 9 | 498 | 0.516 |
| 10 | 495 | 0.429 |
| 11 | 490 | 0.345 |
| 12 | 495 | 0.303 |
| 13 | 492 | 0.271 |
| 14 | 488 | 0.293 |
| 15 | 495 | 0.239 |
| 16 | 492 | 0.302 |
| 17 | 490 | 0.263 |
| 18 | 495 | 0.229 |
| 19 | 488 | 0.115 |
| 20 | 496 | 0.106 |
| 21 | 498 | 0.102 |
| 22 | 485 | 0.078 |
| 23 | 492 | 0.068 |
| 24 | 498 | 0.061 |
| 25 | 490 | 0.059 |
| 26 | 500 | 0.037 |
| 27 | 492 | 0.029 |
| 28 | 485 | 0.024 |
| 29 | 495 | 0.021 |
| 30 | 492 | 0.013 |

Sample 2 （D=1.4）

| Time/Day | Total volume of leachate/ml | Uranium concentration/g/l |
| --- | --- | --- |
| 1 | 491 | 0.851 |
| 2 | 485 | 1.143 |
| 3 | 485 | 2.094 |
| 4 | 490 | 1.401 |
| 5 | 491 | 0.982 |
| 6 | 485 | 0.843 |
| 7 | 485 | 0.812 |
| 8 | 491 | 0.146 |
| 9 | 490 | 0.709 |
| 10 | 491 | 0.684 |
| 11 | 492 | 0.632 |
| 12 | 490 | 0.582 |
| 13 | 488 | 0.512 |
| 14 | 492 | 0.409 |
| 15 | 489 | 0.325 |
| 16 | 488 | 0.304 |
| 17 | 490 | 0.246 |
| 18 | 493 | 0.211 |
| 19 | 490 | 0.183 |
| 20 | 495 | 0.165 |
| 21 | 492 | 0.155 |
| 22 | 488 | 0.137 |
| 23 | 490 | 0.101 |
| 24 | 492 | 0.092 |
| 25 | 491 | 0.068 |
| 26 | 492 | 0.054 |
| 27 | 491 | 0.033 |
| 28 | 492 | 0.028 |
| 29 | 489 | 0.022 |
| 30 | 492 | 0.018 |

Sample 3 （D = 1.7）

| Time/Day | Total volume of leachate/ml | Uranium concentration/g/l |
| --- | --- | --- |
| 1 | 490 | 0.901 |
| 2 | 490 | 1.681 |
| 3 | 485 | 2.241 |
| 4 | 490 | 1.882 |
| 5 | 491 | 1.426 |
| 6 | 488 | 1.125 |
| 7 | 490 | 0.934 |
| 8 | 491 | 0.826 |
| 9 | 485 | 0.734 |
| 10 | 490 | 0.687 |
| 11 | 488 | 0.631 |
| 12 | 490 | 0.601 |
| 13 | 482 | 0.576 |
| 14 | 490 | 0.532 |
| 15 | 486 | 0.499 |
| 16 | 489 | 0.358 |
| 17 | 492 | 0.288 |
| 18 | 488 | 0.261 |
| 19 | 493 | 0.213 |
| 20 | 490 | 0.201 |
| 21 | 491 | 0.189 |
| 22 | 492 | 0.173 |
| 23 | 488 | 0.162 |
| 24 | 492 | 0.142 |
| 25 | 492 | 0.123 |
| 26 | 489 | 0.102 |
| 27 | 492 | 0.096 |
| 28 | 489 | 0.088 |
| 29 | 490 | 0.064 |
| 30 | 492 | 0.042 |

Sample 4 （D = 2.0）

| Time/Day | Total volume of leachate/ml | Uranium concentration/g/l |
| --- | --- | --- |
| 1 | 490 | 0.951 |
| 2 | 490 | 1.874 |
| 3 | 486 | 2.412 |
| 4 | 493 | 1.912 |
| 5 | 490 | 1.532 |
| 6 | 485 | 1.241 |
| 7 | 495 | 1.035 |
| 8 | 496 | 0.984 |
| 9 | 493 | 0.862 |
| 10 | 489 | 0.723 |
| 11 | 493 | 0.651 |
| 12 | 488 | 0.523 |
| 13 | 495 | 0.501 |
| 14 | 493 | 0.486 |
| 15 | 490 | 0.423 |
| 16 | 488 | 0.409 |
| 17 | 493 | 0.382 |
| 18 | 490 | 0.361 |
| 19 | 495 | 0.321 |
| 20 | 488 | 0.282 |
| 21 | 498 | 0.241 |
| 22 | 491 | 0.203 |
| 23 | 494 | 0.186 |
| 24 | 493 | 0.162 |
| 25 | 487 | 0.124 |
| 26 | 492 | 0.103 |
| 27 | 491 | 0.092 |
| 28 | 482 | 0.086 |
| 29 | 489 | 0.073 |
| 30 | 490 | 0.051 |

Sample 5 （D = 2.3）

| Time/Day | Total volume of leachate/ml | Uranium concentration/g/l |
| --- | --- | --- |
| 1 | 488 | 0.975 |
| 2 | 486 | 1.923 |
| 3 | 490 | 2.489 |
| 4 | 490 | 1.956 |
| 5 | 495 | 1.687 |
| 6 | 488 | 1.321 |
| 7 | 495 | 1.109 |
| 8 | 492 | 0.991 |
| 9 | 493 | 0.872 |
| 10 | 490 | 0.731 |
| 11 | 495 | 0.661 |
| 12 | 487 | 0.584 |
| 13 | 493 | 0.531 |
| 14 | 488 | 0.501 |
| 15 | 490 | 0.478 |
| 16 | 496 | 0.456 |
| 17 | 488 | 0.412 |
| 18 | 493 | 0.385 |
| 19 | 486 | 0.342 |
| 20 | 494 | 0.301 |
| 21 | 496 | 0.263 |
| 22 | 490 | 0.212 |
| 23 | 491 | 0.195 |
| 24 | 487 | 0.164 |
| 25 | 489 | 0.123 |
| 26 | 489 | 0.098 |
| 27 | 488 | 0.075 |
| 28 | 490 | 0.065 |
| 29 | 490 | 0.056 |
| 30 | 492 | 0.049 |

Sample 6 （D = 2.6）

| Time/Day | Total volume of leachate/ml | Uranium concentration/g/l |
| --- | --- | --- |
| 1 | 490 | 0.981 |
| 2 | 492 | 1.943 |
| 3 | 490 | 2.53 |
| 4 | 490 | 1.982 |
| 5 | 490 | 1.701 |
| 6 | 485 | 1.456 |
| 7 | 491 | 1.123 |
| 8 | 493 | 0.998 |
| 9 | 490 | 0.881 |
| 10 | 492 | 0.736 |
| 11 | 488 | 0.672 |
| 12 | 490 | 0.601 |
| 13 | 487 | 0.561 |
| 14 | 492 | 0.512 |
| 15 | 488 | 0.489 |
| 16 | 495 | 0.461 |
| 17 | 492 | 0.423 |
| 18 | 488 | 0.391 |
| 19 | 492 | 0.321 |
| 20 | 492 | 0.306 |
| 21 | 491 | 0.271 |
| 22 | 488 | 0.251 |
| 23 | 490 | 0.221 |
| 24 | 491 | 0.199 |
| 25 | 490 | 0.162 |
| 26 | 489 | 0.132 |
| 27 | 493 | 0.112 |
| 28 | 490 | 0.092 |
| 29 | 488 | 0.075 |
| 30 | 487 | 0.061 |
